# Transcriptional regulation of FACT involves Coordination of chromatin accessibility and CTCF binding

**DOI:** 10.1016/j.jbc.2023.105538

**Published:** 2023-12-10

**Authors:** Peijun Wang, Na Fan, Wanting Yang, Pengbo Cao, Guojun Liu, Qi Zhao, Pengfei Guo, Xihe Li, Xinhua Lin, Ning Jiang, Buhe Nashun

**Affiliations:** 1 Inner Mongolia Key Laboratory for Molecular Regulation of the Cell, Inner Mongolia University, Hohhot, China; 2 State Key Laboratory of Reproductive Regulation and Breeding of Grassland Livestock, School of Life Sciences, Inner Mongolia University, Hohhot, China; 3 School of Life Science and Technology, Inner Mongolia University of Science and Technology, Baotou, China; 4 State Key Laboratory of Genetic Engineering, School of Life Sciences, Fudan University, Shanghai, China; 5 Inner Mongolia Saikexing Institute of Breeding and Reproductive Biotechnology in Domestic Animals, Hohhot, China

**Keywords:** histone chaperone, FACT, Ssrp1, chromatin accessibility, CTCF, transcription

## Abstract

Histone chaperone FACT (facilitates chromatin transcription) is well known to promote chromatin recovery during transcription. However, the mechanism how FACT regulates genome-wide chromatin accessibility and transcription factor binding has not been fully elucidated. Through loss-of-function studies, we show here that FACT component Ssrp1 is required for DNA replication and DNA damage repair and is also essential for progression of cell phase transition and cell proliferation in mouse embryonic fibroblast cells. On the molecular level, absence of the Ssrp1 leads to increased chromatin accessibility, enhanced CTCF binding, and a remarkable change in dynamic range of gene expression. Our study thus unequivocally uncovers a unique mechanism by which FACT complex regulates transcription by coordinating genome-wide chromatin accessibility and CTCF binding.

In eukaryotes, nucleosome is the basic unit of chromatin, which consists of approximately 146-bp DNA wrapped around a histone octamer (1, 2). DNA is packaged into nucleosomes to ensure the integrity of genome, which at the same time sets a barrier for DNA-related processes. Consequently, nucleosome dynamics mediated by histone assembly and disassembly needs to be finely regulated to ensure normal genomic functions, where histone chaperones and chromatin remodelers are actively involved.

FACT (facilitates chromatin transcription) is an essential histone chaperone consisting of two subunits, Spt16 (suppressor of Ty homolog 16) and Ssrp1 (structure-specific recognition protein 1) (3), both of which are highly conserved in eukaryotes (4, 5). FACT regulates chromatin homeostasis in DNA-centered processes including transcription, DNA replication, and DNA repair (3, 6). It has been shown that FACT associates with actively transcribed genes *in vivo* and facilitates passage of the RNA polymerase Ⅱ during transcription by displacing H2A/H2B from nucleosome (7). However, recent studies suggested that FACT maintains nucleosome integrity through tethering all components of the nucleosome together (8). FACT also plays a role in suppressing cryptic transcription (9, 10), silencing heterochromatin (11, 12), and inhibiting expression of subtelomeric genes and MERVL retrotransposon (13, 14, 15, 16). Intriguingly, FACT is abundantly expressed in undifferentiated or tumor cells, while it has very limited expression in differentiated cells, suggesting possible involvement in maintenance of undifferentiated cell status (17, 18, 19). Substantial progress has been made in understanding the structural features of FACT and its roles in development and carcinogenesis (20, 21, 22). However, the molecular mechanism how FACT regulates gene expression through coordinating chromatin accessibility in a genome-wide context has not been fully elucidated.

Gene expression is controlled at multiple layers, of which the three-dimensional (3D) organization of chromosomes enables cells to balance spatial constraints of nucleus with the functional dynamics of gene regulation (23). Topologically associating domains (TADs) are important contexts of 3D genome organization that are separated by insulating boundaries enriched with architectural proteins (24). CCCTC-binding factor (CTCF) is an 11-zinc-finger, insulator-binding protein that induces chromatin looping and binding at TAD boundaries (25). CTCF and cohesion are the main contributors for the formation of TADs, whose binding characteristics at the border of TADs are well conserved in different cell types (26). Numerous studies have demonstrated the functional importance of CTCF in regulation of gene expression, showing that inversion, deletion, mutation, or mispositioning of CTCF is sufficient to impair high-order chromatin interactions and transcriptional regulation (27, 28, 29, 30, 31). However, the interplay between FACT-mediated chromatin accessibility, CTCF binding, and transcription regulation has been rarely reported.

In this study, we investigated the regulatory role of FACT in chromatin accessibility and transcription by combining assay for transposase-accessible chromatin with high-throughput sequencing (ATAC-seq), RNA-seq, and CUT&Tag approaches. CRISPR-Cas9-mediated ablation of Ssrp1, the smaller subunit of FACT complex, increased overall chromatin accessibility and greatly elevated CTCF binding, leading to compromised gene expression dynamics in mouse embryonic fibroblast cells. Ssrp1 is also required for DNA replication, DNA damage repair, cell cycle progression, and cell proliferation. Therefore, we proposed a previously unrecognized role of the histone chaperone FACT whereby FACT regulates gene expression through coordinating genome-wide chromatin accessibility and CTCF binding, which in turn safeguards normal cellular functions.

## Results

### Ssrp1 is enriched around TSS and abundantly bound to promoters

**Figure 1:**
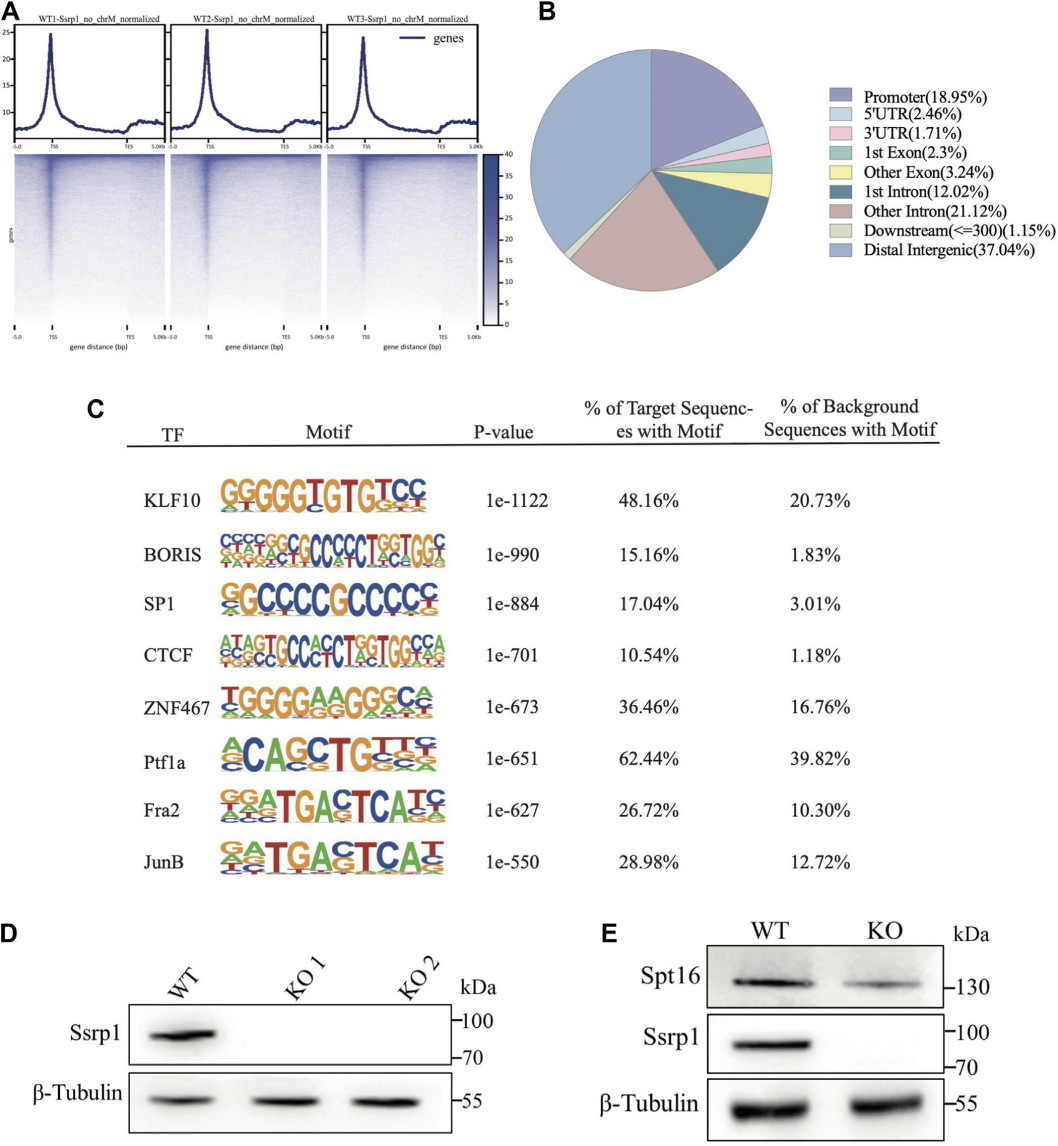
**Ssrp1 is enriched around TSS and abundantly bound to promoters.***A*, heatmap showing Ssrp1 CUT&Tag peaks enriched around the TSS. *B*, pie chart of the genomic location distribution of Ssrp1, showing the percentage for each genomic location category. *C*, transcription factor (TFs) motif enrichment analysis showing Ssrp1 binding regions (identified by CUT&Tag) are also enriched with KLF10, BORIS, SP1, CTCF, ZNF467, Ptf1a, Fra2, and JunB binding motifs *D*, Western blot analysis of Ssrp1 protein in WT and two different Ssrp1-KO cell clones. *E*, Western blot analysis of Spt16 and Ssrp1 protein in WT and Ssrp1-KO cells. β-Tubulin was used as loading control in *D* and *E*.

In order to investigate the potential impact on transcription regulation, CUT&Tag-sequencing (CUT&Tag-seq) (32) of Ssrp1, the smaller subunit of FACT complex, was performed in three independent biological replicates of mouse embryonic fibroblast (MEF) cells to investigate the genome-wide binding preference. A total of 18,153 Ssrp1 peaks were detected throughout the genome (Fig. S1*C*), and strong enrichment of Ssrp1 around the transcription start site (TSS) was observed (Fig. 1*A*). Moreover, Ssrp1 was also abundantly bound in the promoters, accounting for approximately 19% of the peaks (Fig. 1*B*). We further performed *de novo* motif analysis in Ssrp1 CUT&Tag-seq peaks, followed by scanning for matching known transcription factor (TF) binding preferences to examine whether the chromatin regions enriched with Ssrp1 binding are also enriched with other TFs binding motifs. It was found that motifs matching binding preferences for GC-rich transcription factors such as KLF10, BORIS, SP1, and CTCF were enriched at Ssrp1-binding sites, indicating that Ssrp1-enriched regions might subject to collaborative regulation of several transcription factors and play an important role in overall transcription regulation (Fig. 1*C*).

In order to unambiguously define the role of FACT in transcriptional regulation, we set out to delete the Ssrp1 by CRISPR-Cas9 method. Given that the HMG domain of Ssrp1 is essential for FACT to reorganize nucleosomes (33), guide RNA (gRNA) targeting the HMG domain of Ssrp1 in the exon 14 region was designed (Fig. S1*A*) (3, 34, 35). Two cell clones with Ssrp1 knockout (Ssrp1-KO) were obtained (see also Experimental procedures), and CRISPR-induced mutations were confirmed using Sanger sequencing, of which one clone includes a 7-bp and the other includes a 20-bp deletion in the exon 14 (Fig. S1*B*). Western blotting analysis further confirmed absence of the Ssrp1 protein in the two cell clones (Fig. 1*D*) (32). Moreover, expression of Spt16, the other subunit of FACT, was dramatically reduced but not completely eliminated in the Ssrp1-KO cells (Fig. 1*E*), which is consistent with previous report in human cells that the protein stability of each subunit of FACT depends on the presence of the other subunit and the presence of Ssrp1 mRNA is critical for Spt16 protein stability (36). We further examined 14 potential off-target sites that contain 13 to 17 identical nucleotides to the gRNA and confirmed no mutations occurred at these sites (Table S1*A*). Taken together, these results showed that Ssrp1 was successfully depleted in mouse embryonic fibroblasts, which impair the stability of Spt16 subunit and disrupts the function of the FACT complex. Both the KO cell clones were used alternatively in the following experiments.

Considering the ability of FACT to promote H2A–H2B deposition (34, 37, 38, 39, 40), we used H2A CUT&Tag approach to examine change in H2A content after Ssrp1 depletion. Since H2A is abundantly distributed all over the genome, it is technically difficult to detect all H2A peaks. Therefore, we focused on comparing and identifying changes in H2A peaks in the wildtype (WT) and KO cells. In agreement with our prediction, overall H2A CUT&Tag signal was dramatically decreased in the Ssrp1-KO cells (Fig. S1, *D* and *E*) and the signal in all genomic elements, especially in promoter regions, was markedly reduced (Fig. S1*E*). Consistently, immunoblotting with acid-extracted histone H2B showed that H2B content was significantly reduced in the Ssrp1-KO cells (Fig. S1*F*).

### Ssrp1 depletion resulted in severe cellular phenotype, associated with DNA replication, DNA damage, cell cycle progression, and cell proliferation

**Figure 2:**
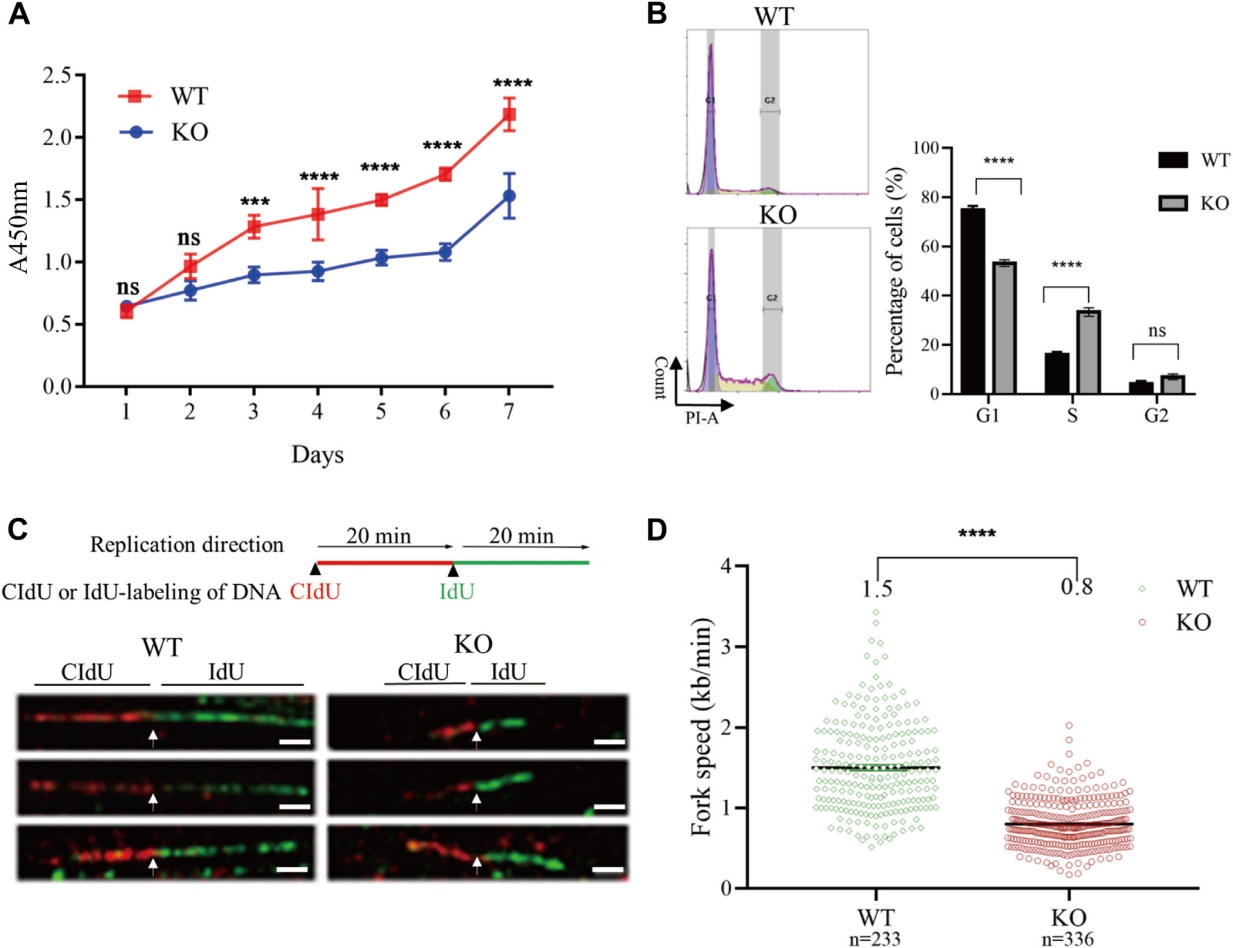
**Ssrp1 depletion resulted in severe cellular phenotype, associated with DNA replication, DNA damage, cell cycle progression, and cell proliferation.***A*, CCK8 analysis of cell proliferation in the WT and Ssrp1-KO cells. The data were obtained from three independent experiments and presented as mean ± SEM. Statistical analysis was conducted by the two-way ANOVA test. *B*, flow cytometry analysis of cell cycle in the WT and Ssrp1-KO cells. DNA was stained with propidium iodide. *C*, representative DNA fibers images. *Upper panel*: Schematic illustration of the DNA fiber assay. Cells were pulse-labeled with chlorodeoxyuridine (CIdU) (*red*) and iododeoxyuridine (IdU) (*green*) for 20 min. *Lower panel*: three representative images of typical DNA fibers obtained from WT or Ssrp1-KO cells. The scale bar represents 2 μm. *D*, FACT is required for DNA replication fork progression. The lengths of the DNA fibers were measured, and the fork rates were calculated as fibers length divided by pulse labeling time. *p* Values were calculated by the Mann–Whitney U test. N represents the number of measured DNA fibers. One representative result of three independent experiments is shown. ns, nonsignificant, ∗∗∗ *p* < 0.001 or ∗∗∗∗ *p* < 0.0001.

Evaluation of Ssrp1-depleted MEF cells showed that loss of Ssrp1 resulted in a severe cellular phenotype. Deletion of Ssrp1 significantly inhibited proliferation of MEF cells (Fig. 2*A*). Moreover, Flow cytometry analysis showed that absence of Ssrp1 lead to a dramatic decrease of cells in the G1 phase while a significant increase of cells in the S phase (Fig. 2*B*). Given that FACT-mediated nucleosome assembly is essential for DNA replication (41, 42), we speculated that the observed S phase delay in Ssrp1-KO cells may be due to defect in DNA replication. Since normal progression of replication fork depends on efficient nucleosome assembly behind the fork (43), we examined the effect of FACT depletion on replication fork progression by single-molecule analysis of stretched DNA fibers. The cells were consecutively pulse labeled with chlorodeoxyuridine (CIdU) and iododeoxyuridine (IdU) for 20 min, respectively, and the lengths of the labeled DNA fiber tracks were measured by immunostaining (Fig. 2*C*). Quantification analysis showed that the speed of DNA replication fork progression was significantly reduced in the Ssrp1-KO cells and the average speed was only about half that of the WT cells (Fig. 2*D*). Consistently, transcriptome analysis showed that the hexameric DNA helicase MCM2-7 was significantly downregulated by the loss of Ssrp1 (Fig. S2*A*). These data collectively suggested that Ssrp1 is required for normal replication fork progression and the delayed S phase progression observed in Ssrp1-KO cells was due to slower DNA replication fork progression, at least partially.

Since proper chromatin organization is essential for protecting DNA from damage (44) and FACT plays an important role in maintaining chromatin stability, we set out to examine the level of DNA double-strand break by its marker γ-H2A.X in WT and Ssrp1-KO cells. Western blotting showed that Ssrp1-KO cells had increased levels of γ-H2A.X (Fig. S2*B*), suggesting that Ssrp1 may play a role in prevention of the spontaneous DNA damage. In order to further examine whether Ssrp1 is involved in DNA repair process, the cells were treated with H_2_O_2_ for 30 min and γ-H2A.X level was examined at 15, 30, 60, 120, and 240 min after removal of H_2_O_2_ (Fig. S2*C*). γ-H2A.X level was gradually increased and reached the highest level after 30 min recovery both in the WT and KO cells, then gradually declined in the WT cells but sustained high in the KO cells (Fig. S2*C*), suggesting that absence of Ssrp1 impaired DNA repair. In support of this view, DNA damage response genes were significantly enriched among the genes expressed in the Ssrp1-KO cells (Fig. S2*D*) and Ssrp1 deletion remarkably reduced the expression of a number of DNA damage repair genes, including *Mre11a*, *Parp1*, *Pcna*, *Rad54*, and *Rfc2* (Fig. S2*E*). Taken together, these data suggested that Ssrp1 is required for DNA replication and DNA damage repair and eventually required for cell cycle progression and cell proliferation in mouse embryonic fibroblast cells.

### FACT is necessary to maintain the full dynamic range of gene expression

**Figure 3:**
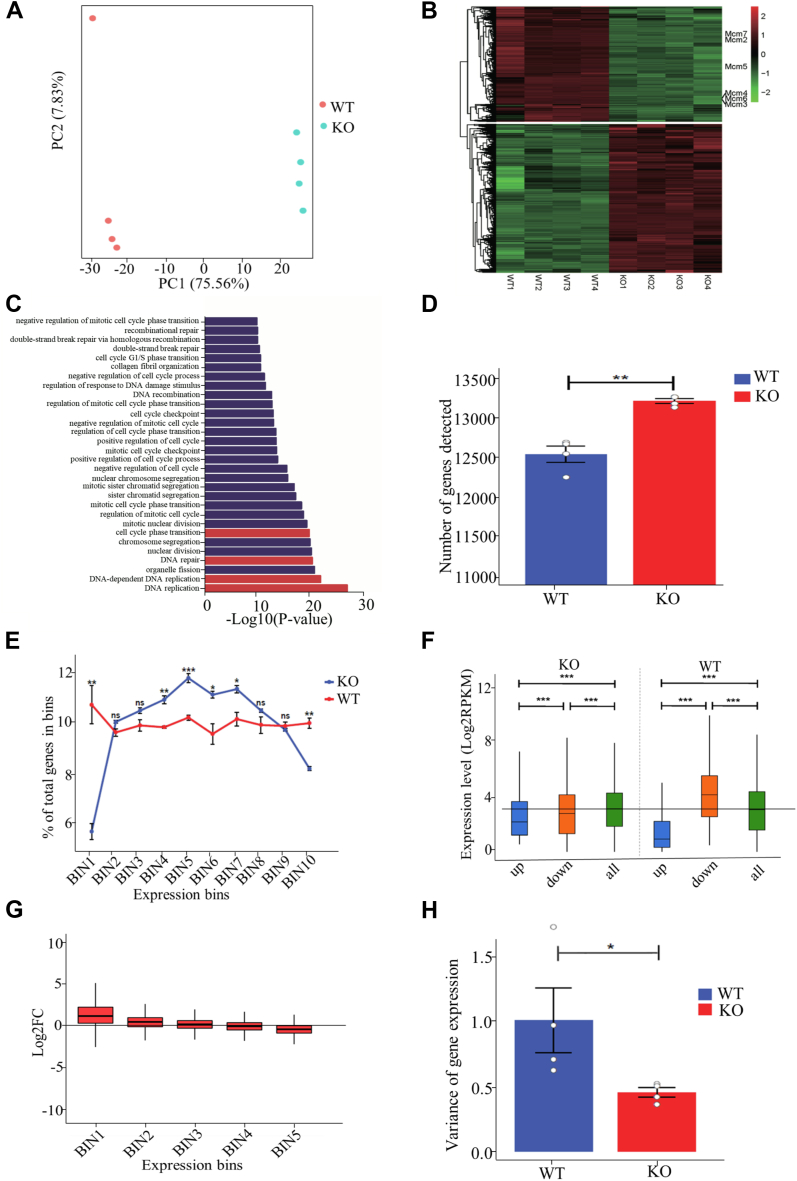
**Ssrp1 was necessary to maintain full dynamic range of gene expression.***A*, principal component analysis (PCA) of RNA-seq data derived from WT and Ssrp1-KO cells. The samples are represented by different colors as indicated in the *right*. *B*, heatmap of differentially expressed genes between WT and Ssrp1-KO cells. Each row represents a gene, and different colors represent expression levels. *C*, the top 30 gene ontology terms significantly enriched among the differentially expressed genes in the Ssrp1-KO cells. The biological processes associated with the observed cellular phenotype in the Ssrp1-KO cells are highlighted. *D*, number of annotated genes as computed by HTSeq program is shown. *E*, relative proportion of genes distributed among ten equally sized expression level bins. All genes detected in our RNA-seq experiment were divided into ten equal bins based on their expression levels in WT cells from *low to high*. The gene number in each bin was counted for WT or Ssrp1-KO, and the percentage was calculated relative to all genes detected in a given sample. *F*, boxplot shows gene expression levels of differentially upregulated (edgeR, false discovery rate <0.1), downregulated (edgeR, false discovery rate <0.1), and all annotated genes in WT and Ssrp1-KO cells. *G*, boxplots show distribution of gene expression fold change for each gene expression level quintile (based on WT sample). *H*, variance of gene expression within a given WT or Ssrp1-KO sample. In all cases, error bars indicate SEM. Statistical analysis was carried out by two-tailed unpaired Student’s *t* test (*D* and *E*), Kruskal–Wallis with Dunn’s *post hoc* test (*F*), or F test (*H*); ns, nonsignificant; ^∗^*p* < 0.05, ^∗∗^*p* < 0.01, and ^∗∗∗^*p* < 0.001.

In order to uncover the underlying mechanism of the observed cellular phenotypes (Figs. S2 and 2) and investigate the role of FACT in transcription regulation, we performed transcriptome analysis using four replicates each of the WT and Ssrp1-KO cells. Principal component analysis revealed a remarkable transcriptional difference upon depletion of the Ssrp1 (Fig. 3*A*). Further analysis identified 3931 differentially expressed genes (*p*-value ＜ 0.05 and |log2FoldChange| > 1; Figs. 3*B* and S3*A*), among which upregulated genes (2214 genes) were 29% more than the downregulated genes (1717 genes). In agreement with the severe cellular phenotypes (Fig. 2), Gene Ontology (GO) analysis of differentially expressed genes showed significant enrichment in biological processes related to DNA replication, DNA repair, and cell phase transition (Fig. 3*C*). Kyoto Encyclopedia of Genes and Genomes (KEGG) analysis of differentially expressed genes revealed significantly changed biological pathways, including DNA replication, cell cycle, and multiple DNA repair signaling pathways (Fig. S3, *B* and *C*). These results collectively suggested that the FACT complex regulates cellular function through overseeing DNA-templated processes.

Intriguingly, more genes were detected in Ssrp1-KO cells than WT cells (Fig. 3*D*), suggesting that FACT may function to prevent inappropriate transcriptional initiation (15). The increased number of transcripts was not evenly distributed across all expression levels (Fig. 3*E*); rather, deletion of Ssrp1 resulted in relatively fewer transcripts with very low (bin 1) or very high (bin 10) expression (Fig. 3*E*) and a significant increase in the proportion of genes with moderate expression levels (bin 5). In addition, differentially up- and downregulated genes showed lower than expected expression (Fig. 3*F*). Moreover, while the genes normally not expressed or expressed at very low levels (Fig. 3*G*, bin 1 and bin 2) were upregulated upon Ssrp1 deletion, genes with very high expression (Fig. 3*G*, bin 5) were specifically downregulated in Ssrp1-KO cells. Notably, loss of Ssrp1 led to greatly reduced expression variance of transcripts (Fig. 3*H*). Collectively, these results suggested that Ssrp1 was necessary for the full dynamic range of gene expression.

### FACT deficiency increases chromatin accessibility

**Figure 4:**
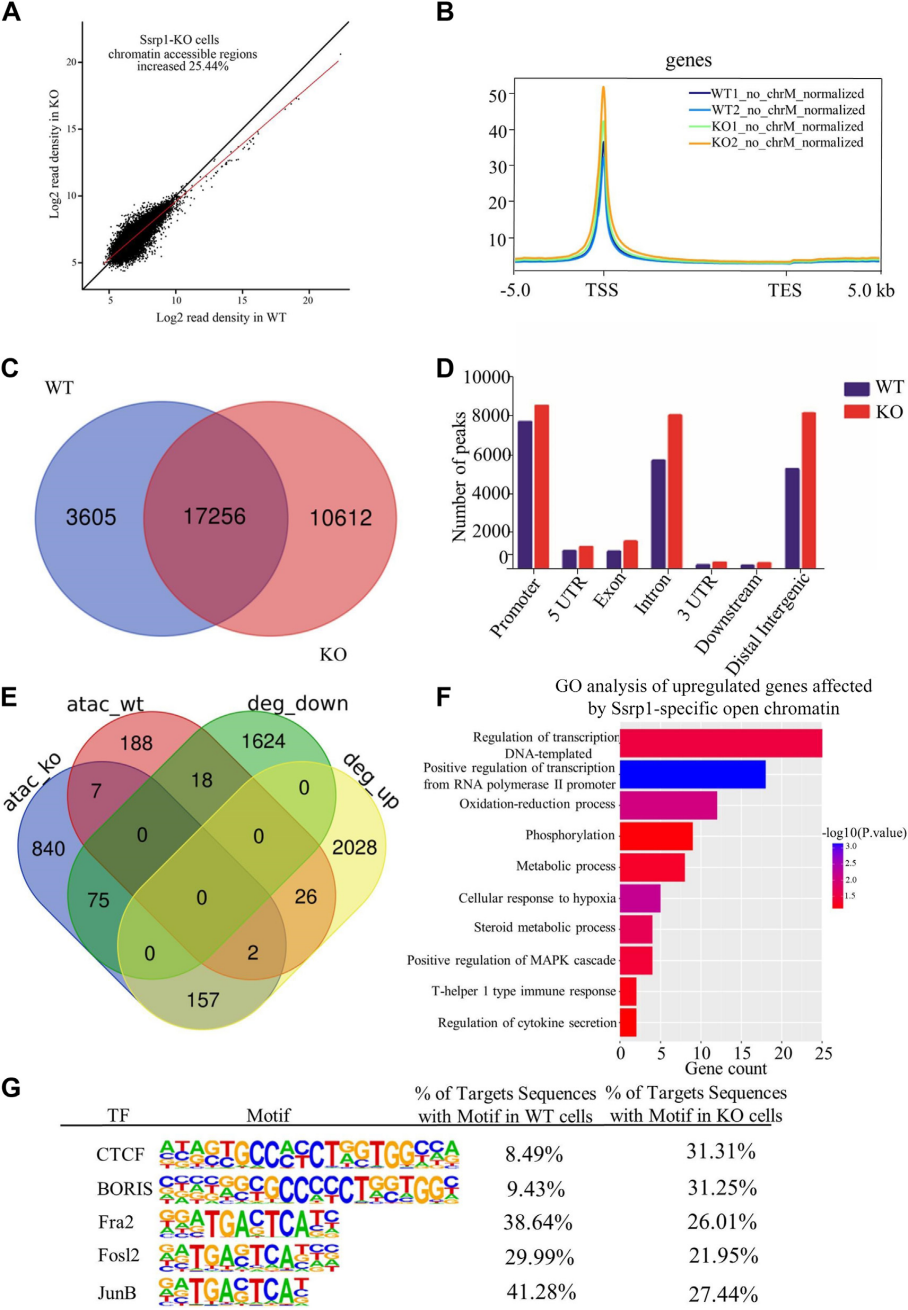
**Absence of Ssrp1 increased genome-wide chromatin accessibility.***A*, comparison of the normalized ATAC-seq coverage density in WT and Ssrp1-KO cells revealed holistic upregulation of the chromatin accessibility in the Ssrp1-KO cells. The *x*-axis represents the log2 read density of all detected peaks in WT, and the *y*-axis represents the log2 read density of the corresponding peaks in KO. The *red* diagonal line represents the *y*=*x* axis, assuming no global chromatin accessible changes in WT and Ssrp1-KO cells; the *black* diagonal line represents the best linear fitting line for the coverage density of Ssrp1-KO *versus* WT. *B*, average ATAC-seq peak density ±5 kb of the TSS and TES. *C*, Venn diagram shows the unique or common ATAC-seq peaks of the WT and Ssrp1-KO cells. *D*, histogram shows genomic distribution of ATAC-seq peaks in the WT and Ssrp1-KO cells. Each bar represents the number of peaks in different genomic contexts. *E*, Venn diagram shows the intersection of differentially expressed genes and genes that were annotated by the ATAC-seq peaks in the promoter regions; deg, differentially expressed genes. *F*, top 10 gene ontology (GO) terms of the genes that have upregulated gene expression and increased chromatin accessibility after Ssrp1 deletion. *G*, HOMER motif analysis shows significant enrichment of the CTCF binding motif in the Ssrp1-KO cells. Top five most enriched transcription factor binding motifs in the Ssrp1-KO cells and their proportions in the Ssrp1-KO and WT cells are shown. TES, transcription end site; TSS, transcription start site.

Since FACT exerts its gene regulatory effect through nucleosome reorganization (3, 22, 45), we asked whether Ssrp1 disruption alters chromatin accessibility. Therefore, we performed ATAC-seq analysis using two biological replicates of WT and Ssrp1-KO cells (Fig. 4). Consistent with our expectation, initial analysis revealed an average increase of approximately 25% in genome-wide chromatin accessibility upon Ssrp1 depletion (Fig. 4*A*). The chromatin accessible sites were enriched around transcription start sites, and the average signal in the Ssrp1-KO cells was higher than that of WT cells (Figs. 4*B* and S4*A*). Further analysis identified 20,861 and 27,868 chromatin accessible sites in the WT and Ssrp1-KO cells, respectively (Fig. 4*C*). Among them, 10,612 sites (38%) were unique to Ssrp1-KO cells, indicating that disruption of Ssrp1 made normally not accessible chromatin sites accessible in the KO cells. Apart from the distal intergenic regions, chromatin accessible sites were mainly enriched in genomic regions of the promoters and introns, where the number of ATAC-seq peaks were significantly increased in the Ssrp1-KO cells compared with the control (Fig. 4*D*). Then, we carried out integrated analysis of the RNA-seq and ATAC-seq data to identify genes that were upregulated by the increased chromatin accessibility in the KO cells. A total of 157 genes had upregulated gene expression and increased chromatin accessibility in the promoter regions after Ssrp1 depletion (Fig. 4*E*). These 157 genes were enriched in the biological processes of transcription regulation, oxidation–reduction, and metabolic processes (Fig. 4*F*). Overall, these results suggested that increased chromatin accessibility plays an important role in transcription regulation in the absence of FACT.

In order to examine whether the increased chromatin accessibility after Ssrp1 depletion impacts chromatin binding of transcription factors, we set out to identify TF binding motifs in the open chromatin regions. Motif enrichment assay revealed that the unique open chromatin regions in the Ssrp1-KO cells showed specific binding to a set of TFs, including CTCF, BORIS, Fra2, Fosl2, and JunB (top five of the most enriched transcription factor binding motif). The percentage of the CTCF binding motif in the overall chromatin accessible regions was increased from 8.49% to 31.31% after Ssrp1 deletion (Fig. 4*G*), suggesting that a number of CTCF binding motifs normally not accessible in the WT condition became accessible after Ssrp1 depletion. Notably, the second-ranked TF binding motif, BORIS (Fig. 4*G*), is a paralog of the CTCF (46). Intriguingly, the percentage of the BORIS binding motif in the overall chromatin accessible regions also dramatically increased from 9.43% to 31.25% after Ssrp1 deletion (Fig. 4*G*). In contrast, the percentages of the other TF binding motifs, including Fra2, Fosl2, and JunB, in the overall chromatin accessible regions were reduced remarkably (Fig. 4*G*). Since open chromatin regions possess high GC content relative to the rest of the genome (47), we compared other transcription factors with GC-rich recognition motifs such as Sp1 and Smads in WT and Ssrp1-KO cells. Loss of Ssrp1 also increased the percentage of these transcription factor binding motifs in the overall chromatin accessible regions, but to a much smaller extent than that of the CTCF and BORIS (Fig. S5*E*). Therefore, it is likely that deletion of Ssrp1 provides an overall more open chromatin landscape where the number of accessible CTCF-binding sites was increased mostly and favors CTCF chromatin binding.

### FACT deletion enhances CTCF binding to chromatin

**Figure 5:**
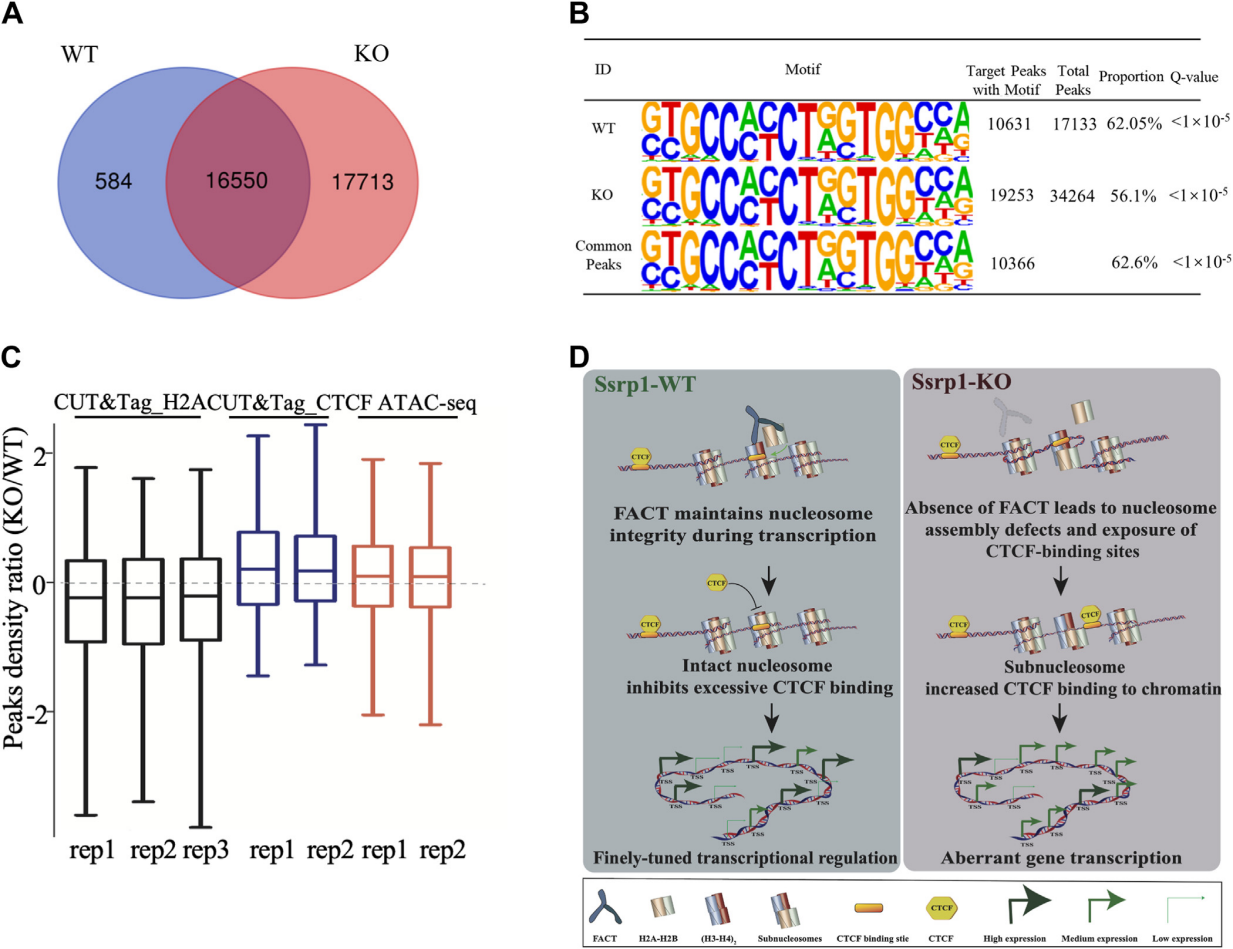
**Ssrp1 deletion enhances CTCF binding to chromatin.***A*, Venn diagram shows the unique and common CTCF CUT&Tag peaks of the WT and Ssrp1-KO cells. *B*, motif analysis of the CTCF CUT&Tag peaks in the WT and Ssrp1-KO cells. *C*, peak density ratio boxplot of CUT&Tag_H2A, CUT&Tag_CTCF, and ATAC-seq. *D*, working model for FACT fine-tunning transcriptional regulation by coordinating subnucleosome, chromatin accessibility, and CTCF binding.

In order to investigate whether the increased chromatin accessibility and more accessible CTCF binding motifs promote CTCF binding to chromatin, we performed CUT&Tag analysis of CTCF, which has lower signal-to-noise ratio than chromatin immunoprecipitation with sequencing (32). Two biological replicates of WT or Ssrp1-KO cells showed good consistency (Fig. S5, *A* and *B*). In agreement with previous reports (48), heatmap analysis showed that the CTCF binding peaks were enriched around TSS regions (−0.5 to +0.5 kb from TSS) (Fig. S5*C*), which is also very similar to the distribution feature of the open chromatin regions identified by the ATAC-seq (Fig. S4*A*). A total of 17,134 and 34,263 CTCF binding peaks were identified in the WT cells and Ssrp1-deficient cells, respectively (Fig. 5*A*), showing that loss of Ssrp1 resulted in twice the number of CTCF binding to chromatin in the Ssrp1-KO cells than in control cells. Among them, 16,550 were represented in both the WT and Ssrp1-KO cells, 584 (3.4%) were unique to WT cells, and 17,713 (51.7%) were unique to Ssrp1-KO cells (Fig. 5*A*). This result indicates that CTCF binding in the WT cells was almost maintained after Ssrp1 depletion, while CTCF binding to new chromatin regions was greatly enhanced after Ssrp1 depletion. This increased CTCF binding was observed at the whole-genome level, particularly at the promoters, introns, and distal intergenic regions (Fig. S5*D*).

Further analysis identified 10,631 and 19,253 CTCF binding peaks within the CTCF binding motifs in the WT and Ssrp1-KO cells, respectively, accounting for approximately 62.05% of the total CTCF binding peaks in the WT cells and 56.01% in Ssrp1-KO cells (Fig. 5*B*). Notably, 10,366 of them were common to both types of the cells, only 265 (2.4%) peaks were unique to WT, *versus* 8987 (46.7%) peaks were unique in the KO cells. This finding shows that CTCF binding to the CTCF binding motifs almost doubled in the Ssrp1-KO cells, mainly in the normally not accessible CTCF binding motifs (Fig. 5*B*). Taken together, these results collectively demonstrated that Ssrp1 deletion increased overall CTCF binding on chromatin, with remarkable increase in the CTCF binding motifs.

Integrated analysis of H2A CUT&Tag, CTCF CUT&Tag, and ATAC-seq data showed that global H2A occupancy was reduced in Ssrp1 KO cells while genome-wide chromatin accessibility and CTCF binding were increased (Fig. 5*C*). Taken together, these data collectively suggested that loss of FACT leads to nucleosome assembly defects and increases chromatin accessibility, which in turn leads to exposure of normally not accessible CTCF binding motifs and enhances CTCF binding. All these changes in chromatin result in aberrant transcriptional regulation in the Ssrp1-depleted cells. Therefore, we propose here that the histone chaperone FACT maintains full dynamic range of transcription by regulating chromatin accessibility and CTCF binding (Fig. 5*D*).

## Discussion

In this study, we demonstrated that genetic deletion of Ssrp1, the smaller subunits of histone chaperone FACT, dramatically increased global chromatin accessibility and CTCF binding. Absence of Ssrp1 abolishes FACT-mediated chromatin homeostasis and disrupts transcriptional regulation, which results in pronounced cellular phenotypes including cell cycle blockage and reduced cell proliferation. Different from existing studies focusing on FACT-mediated nucleosome dynamics (20, 21, 37), we propose a transcriptional regulatory mechanism whereby FACT regulates genome-wide chromatin accessibility and CTCF binding to chromatin.

FACT binds multiple components of nucleosome and keeps them together to prevent dispersing and facilitate nucleosome reassembly (3). This property of FACT is essential for maintaining chromatin homeostasis during cellular processes, such as DNA replication and repair (49). Our results documented that depletion of Ssrp1 in MEF cells leads to defects in DNA replication, DNA damage repair, cell cycle progression, and cell proliferation (Fig. 2). The delay in S phase observed in Ssrp1-KO cells (Fig. 2*B*) suggested that FACT is necessary for DNA replication. FACT has been reported to cooperate with CAF-1 and Rtt106 during replication-coupled nucleosome assembly in yeast (42). Failure to assemble newly synthesized DNA into chromatin slows down replication fork progression in human cells (43), which is in agreement with the slower replication fork progression in the Ssrp1-KO cells (Fig. 2*D*). Furthermore, MCM2-7 expression was significantly reduced after FACT deletion (Fig. S2*A*). Given that the direct physical interaction of FACT and MCM proteins promotes progression of replication fork in HeLa cells (50, 51), it is likely that the decreased expression of the DNA replication helicase in Ssrp1-KO cells is another important reason for the delayed progression of cell cycle in the Ssrp1-KO cells. On the other hand, our experiments documented an increased DNA damage in the absence of Ssrp1 (Fig. S2*B*), which is probably due to the increased chromatin accessibility (Fig. 4*A*). Our data also suggested a possible involvement of Ssrp1 in DNA damage repair (Fig. S2*C*), which is in agreement with previous studies showing that Ssrp1 safeguards genome stability (3, 52, 53). Therefore, defects in DNA replication and increased DNA damage might ultimately impair normal cell cycle progression and cell proliferation. Of note, deletion of Ssrp1 also greatly reduced the level of cell proliferation marker Ki67 (Table S2), which is in line with the decreased cell proliferation in the absence of Ssrp1 (Fig. 2*A*). However, it should be noted that loss of Spt16, the other subunit of the FACT complex, does not affect cell proliferation (54), suggesting the possibility that the two subunits of the FACT complex have unique functions independent of each other (55, 56) and indeed Ssrp1 works together with p63 as a transcriptional coactivator (57).

Previous report in mouse embryonic stem cells found that little changes in nucleosome occupancy was observed after Ssrp1 deletion (16), probably due to the fact that deletion of FACT does not affect chromatin content of histone H3 and H4 (58, 59) but produces more nucleosome hexamers lack of H2A/H2B dimer, also known as subnucleosome (58). Subnucleosomes do not disintegrate and are prevalently within the genome (60, 61). Intriguingly, it has been demonstrated recently that CTCF is generally bound to subnucleosomes (58, 62, 63, 64) and CTCF-bound insulators are highly accessible (65). Our experiments documented that, although FACT lacks the ability to assemble intact nucleosomes, Ssrp1-deleted cells remain transcriptionally active, which results in increased subnucleosome (Figs. 5*C* and S1) and chromatin accessibility (Fig. 4*A*), which in turn lead to enhanced CTCF binding to chromatin (Fig. 5*A*). Consistent with findings in mouse embryonic stem cells (16), we found Ssrp1 is enriched around TSS and promoters in MEF cells (Fig. 1*A*) and chromatin accessibility was increased (Fig. 4*A*) in the absence of Ssrp1, indicating that FACT regulates gene expression by fine-tuning transcription regulatory regions. Based on early *in vitro* studies FACT was termed as a factor facilitating transcription through chromatin (66). However, subsequent FACT inactivation in eukaryotic cells did not result in transcriptional repression (3, 67). Accumulating evidence suggests that FACT maintains nucleosome integrity by tethering all components of the nucleosome together (67, 68, 69) and promotes reassembly of nucleosomes after the RNAPII passage (20, 37, 38, 70). Consistent with previous reports (59, 71), Ssrp1 depletion significantly impaired chromatin content of histone H2A and H2B (Fig. S1, *D*–*F*), and increased chromatin accessibility (Fig. 4*A*), suggesting that FACT plays a crucial role in maintaining nucleosome integrity, which in turn fine-tunes transcription regulation. Therefore, loss of FACT function leads to deficiency in nucleosome assembly and impairs chromatin content, whereby genes can be neither effectively silenced nor activated (Fig. 3, *E* and *G*).

In the context of more accessible chromatin, the number of spurious transcriptions from otherwise not-transcribed regions of the genome increased (Fig. 3*D*). It is noteworthy that our findings revealed an unexpected connection among changes in chromatin accessibility, CTCF binding, and gene transcription. Following Ssrp1 depletion, more accessible chromatin environment leads to a significant increase in the binding of CTCF to chromatin, and this binding is independent of CTCF expression level (Fig. S5*F*). It has been well established that CTCF plays a critical role in organization of chromatin structure and transcription regulation (72). Deletions of insulator CTCF-binding sites cause aberrant chromatin interactions and differential expression of genes within TADs in developmental disorders and cancers (27, 28, 31, 73), and mispositioning of even one CTCF binding locus triggers interactions leading to oncogene activation (28, 73, 74). In our experiments, both increased CTCF binding and aberrant transcriptional regulation were observed following Ssrp1 depletion, suggesting that excessive CTCF binding also leads to transcriptional dysregulation, which may probably be due to aberrant chromatin interactions. In support of this view, it has been reported that increased and aberrant CTCF binding to DNA in acute myeloid leukemia is associated with changed gene expression patterns (75). Furthermore, gene expression variance is greatly reduced in Ssrp1-depleted cells (Fig. 3*H*), which is consistent with the report that increased CTCF binding decreased cell-to-cell variation of gene expression (76). Collectively, our findings documented that histone chaperone FACT could maintain the integrity of nucleosome structure, which in turn is necessary for preventing abnormal CTCF binding and regulation of transcription.

Through Ssrp1 loss of function studies, our results revealed the importance of histone chaperone FACT for the maintenance of chromatin accessibility and CTCF binding, providing novel mechanistic insight into the involvement of FACT in transcription regulation. Since CTCF is a key player for the formation of TAD, it will be interesting to apply Hi-C technology to investigate the molecular mechanism how the increased CTCF chromatin binding alters TAD and impacts gene expression in the absence of FACT.

## Experimental procedures

### Cell culture

The mouse embryonic fibroblast cells line NIH3T3 (SCSP-515) was purchased from the Shanghai Cell Bank, Chinese Academy of Science. The cells were cultured in Dulbecco’s modified Eagle’s medium (Gibco) supplemented with 10% fetal bovine serum (BI), 1% penicillin–streptomycin (Gibco) at 37 °C under 5% CO_2_.

### Generation of Ssrp1 knockout cell lines

The CRISPR-Cas9-mediated Ssrp1 ablation was performed following published protocols (77). In order to induce a gene-inactivating nonsense mutation, several gRNAs for CRISPR-Cas9-mediated Ssrp1 knockout were designed on the CHOPCHOP website (http://chopchop.cbu.uib.no/) and the most effective gRNA was selected: 5′-AAG CCG CGA GAA GAT CAA GT-(PAM)-3′. Target guide sequences were cloned into a BbsⅠ-linearized sgRNA-cloning vector (Addgene #64324) according to published method (78). Briefly, the MEF cells were transfected with 4 μg Ssrp1 gRNA plasmid using Lipofectamine 2000 Transfection Reagent (Invitrogen). Forty-eight hours after recovery, the cells were sorted by flow cytometry and mCherry+ cells were collected and cultured for 6 to 9 days. Monoclonal cell line was obtained by limited dilution method. Briefly, the cells were digested, counted, and diluted to 1 cell/100 μl and seeded into 96-well plates for expansion. Genomic DNA from the individual clones was extracted and used to identify the Ssrp1 gene mutation. Primers flanking the two target Ssrp1 guide sequences were designed to amplify the target fragment containing gRNAs. Target sequence fragments were amplified by polymerase chain reaction (PCR) and sequenced using the following primers: 5′-CCA GGG GAT CTC TTG GAG GA-3′ and 5′- CCC TTC CAG ATC TCC CCT G -3′.

### Off-target analyses in the Ssrp1-KO cell lines

To detect off-target mutations in the Ssrp1-KO cell lines, the Cas-OFFinder software was used to predict potential off-target sites (79), and 14 potential off-target sites were selected (Table. S1*A*), amplified, and sequenced (primers in the Table. S1*B*). The results of multiple sequence alignment showed that no off-target mutations were found at the detection sites.

### Western blot analysis

Cells were lysed in RIPA lysis buffer (P10013B, Beyotime) with protease inhibitor (P1008, Beyotime) and phosphatase inhibitor (P1087, Beyotime). Total protein concentration was measured by the Bradford assay and separated by 12% sodium dodecyl sulfate polyacrylamide gel electrophoresis, then transferred to polyvinylidene fluoride membrane (Millipore). The membrane was blocked with 5% skimmed milk at room temperature (RT) for 1 h and probed with primary antibodies and subsequently incubated with HRP-conjugated secondary antibodies. The antibodies used in this study are listed as follows. Primary antibodies: anti-Ssrp1 (ab137034, Abcam), anti-GAPDH (HC301, TransGen), anti-β-Tublin (66240-1, Proteintech), anti-phospho-Histone H2A.X (ab11175, Abcam), anti-Spt16 (sc-165987, Santa Cruz). Secondary antibodies: goat anti-mouse IgG-HRP (ab97040, Abcam) and goat anti-rabbit IgG-HRP (ab97051, Abcam).

### DNA fiber assay

Cells were pulse-labeled with 25 mM CIdU (Sigma) for 20 min and then sequentially pulse-labeled with 250 mM IdU (Sigma) for 20 min. Cells were resuspended in ice-cold PBS (concentration of 1∗10^3^ cells/μl) and then dropped onto aminopropyl silane-coated glass slides followed by lysis with DNA fiber lysis buffer (0.5% SDS, 200 mM Tris-HCl pH7.4, and 50 mM EDTA). Slides were tilted to extend DNA, and DNA spreads were fixed in methanol/acetic acid (3:1) for 15 min. After washing with PBS, the fiber spreads were treated with HCl to denature DNA molecules. After washing with PBS, the slides were incubated with rat anti-BrdU antibody (detects CIdU, 1:200; Abcam) and mouse anti-BrdU antibody (detects IdU, 1:400; Biolegend) for 1 h and incubated with Cy3-conjugated anti-rat IgG (1:400; Jackson ImmunoReasearch Laboratories) and Alexa Fluor 488 anti-mouse IgG (1:300; Jackson ImmunoReasearch Laboratories) for 1 h. The images were taken under confocal microscopes (Nikon). Fiber lengths were measured using ImageJ. For fork speed analysis, a minimum of 200 fibers were measured per condition during each independent experiment, and micrometer values were expressed in kilobases using the following conversion factor: 1 μm=2.59 kb (80).

### Cell cycle analysis

Cells were fixed in 70% ethanol, stained with propidium iodide, and analyzed by flow cytometry (BD Biosciences). Cell cycle progression was analyzed by the FlowJo software.

### Quantitative RT-PCR

Quantitative RT-PCR assay was performed as described (81). Briefly, total RNAs from cultured cells were extracted with RNAiso Plus (Takara) according to the manufacturer’s instructions and the concentration was determined by a Nanodrop ND-1000 spectrophotometer. The RNA was transcribed into corresponding deoxyribonucleic acid (cDNA) using the PrimeScriptTM RT Reagent Kit with gDNA Eraser (Takara). Each reaction was composed of the following mixture: 1 μl cDNA, 6.25 μl TB Green Premix Taq Ⅱ (2× ), 0.4 μl of each primer, and 4.45 μl ddH_2_O was incubated in LightCycler480 real-time PCR system (Roche). The PCR protocol used was 95 °C for 30 s; 40 cycles of 95 °C for 5 s, and 60 °C for 20 s. All amplifications were done in technical duplicate and biological triplicate, and data were analyzed using LightCycler 96 SW 1.1 software. Primers are listed in the Table. S1*C*.

### RNA-seq and data analysis

Total RNA was extracted from WT or Ssrp1-KO cells using RNAiso Plus according to the manufacture’s instruction (B9109, Takara Bio Inc). Sequencing libraries were constructed using NEBNext Ultra RNA Library Prep Kit (New England Biolabs) for Illumina. Four biological replicates were processed per group. Agilent 2100 bioanalyzer was used for quality control of the sequencing libraries. The libraries were sequenced paired-end 2 × 150 bp using NovaSeq 6000, and each library was sequenced to obtain at least 6 Gb data (Novogene). Adaptor- and quality-trimmed RNA-seq reads were aligned to the mouse genome (Ensembl GRCm38) using Tophat v2.0.8 with default parameters, and only uniquely mapped reads were used to estimate the expression values. Raw counts per gene were obtained using feature Counts. Normalization and differential expressed analysis were performed with the R package DESeq2. The genes with *p*-value less than 0.01 and absolute value of log2 fold change greater than 1 were considered significantly differentially expressed between WT and Ssrp1-KO groups. Volcano plots were generated using R program. DAVID was used to conduct GO (Gene Ontology) and KEGG (Kyoto Encyclopedia of Genes and Genomes) analysis for up- or downregulated genes (https://david.ncifcrf.gov/). Gene set enrichment analysis (GSEA) was performed using GSEA version 4.1.0. Kyng_DNA_Damage_Up gene set was downloaded from the MSigDB database. NES, normalized enrichment score; FDR, false discovery rate (both were calculated in the GSEA program). Total gene expression information based on the mRNA-seq was provided in the Table S2.

### Assay for transposase-accessible chromatin with high-throughput sequencing and data analysis

ATAC-seq was performed as described (82). In brief, 5 × 10^4^ cells were suspended in ice-cold nucleus lysis buffer (10 mM Tris pH 7.4, 10 mM NaCl, 3 mM MgCl_2_, and 0.1% IGEPAL CA-630) for 10 min at 4 °C on a rotation mixer and centrifuged at 500*g* for 10 min at 4 °C. The supernatant was discarded, and the nuclei were then subjected to transposase reaction with Tn5 transposase at 37 °C for 30 min. The digested DNA fragments were purified using MinElute PCR Purification Kit (Qiagen) and analyzed on Bioanalyzer 2100 using High Sensitivity DNA Chip (Agilent 2100). Samples were sequenced on an Illumina HiSeq X Ten platform with 150 PE mode at Frasergen (Frasergen). Two biological replicates were processed per biological sample. ATAC-seq reads were mapped to the mm10 reference sequence. Unmapped or nonuniquely mapped reads along with reads mapped to mitochondria sequence were removed. Only the uniquely mapped reads were used for peak calling analysis. Peak calling was performed using MACS with default parameters. Peaks were annotated with the nearest TSS using ChIPseeker with R. HOMER was performed for motif analysis (83). Total peaks and read density information based on ATAC-seq were provided in Table. S3.

### CUT&Tag experiment and data analysis

CUT&Tag assay was performed as described with minor modifications (32). Briefly, 1 × 10^5^ cells were washed gently twice with wash buffer (20 mM Hepes, pH 7.5; 150 mM NaCl; 0.5 mM Spermidine; 1×Protease inhibitor cocktail) and centrifuged for 5 min at RT. The cell pellets were resuspended in wash buffer. A volume of 10 μl of activated concanavalin A–coated magnetic beads (Bangs Laboratories) was added per sample and incubated at RT for 10 min. The supernatant was removed, and bead-bound cells were resuspended in Dig-wash buffer (20 mM Hepes, pH 7.5; 150 mM NaCl; 0.5 mM Spermidine; 1×Protease inhibitor cocktail; 0.05% Digitonin; 2 mM EDTA). Then, 1 μg of primary antibody (Mouse monoclonal anti-Ssrp1 antibody, 609710, BioLegend; Rabbit monoclonal anti-CTCF antibody, 3418T, CST; Rabbit monoclonal anti-H2A antibody, ab177308, Abcam) was added and incubated on a rotating platform overnight at 4 °C. The primary antibody was removed using magnetic stand. Secondary antibody (Goat anti-Rabbit IgG, B900210, Proteintech, or Rabbit anti-Mouse IgG, ab46540, Abcam) was diluted in Dig-wash buffer (20 mM Hepes, pH 7.5; 150 mM NaCl; 0.5 mM Spermidine; 1×Protease inhibitor cocktail; 0.05% Digitonin), and cells were incubated at RT for 60 min. Cells were washed three times in Dig-wash buffer using the magnetic stand, and unbound antibodies were removed. Hyperactive pA-Tn5 Transposase adapter complex (1:200 dilution) was prepared in Dig-300 buffer (20 mM Hepes, pH 7.5, 300 mM NaCl, 0.5 mM Spermidine, 0.01% Digitonin, 1 × Protease inhibitor cocktail) and incubated with cells at RT for 60 min. Cells were washed with 1 ml Dig-300 buffer for three times. Cells were then resuspended in tagmentation buffer (10 mM MgCl_2_ in Dig-300 buffer) and incubated for 60 min at 37 °C. To stop tagmentation, 10 μl of 0.5 M EDTA, 3 μl of 10% SDS, and 2.5 μl of 20 mg/ml Proteinase K was added to 300 μl of sample, which was incubated for 1 h at 55 °C. DNA was purified using phenol–chloroform–isoamyl alcohol extraction and ethanol precipitation, followed by RNase A treatment. The DNA was amplified by PCR using the following conditions: 72 °C for 3 min, 98 °C for 30 s, 17 cycles of 98 °C for 15 s, 60 °C for 30 s, 72 °C for 30 s, with a final extension at 72 °C for 5 min and hold at 4 °C. Finally, the amplified DNA was purified using Ampure XP Beads (Beckman Counter). Libraries were sequenced 150 bp paired-end on an Illumina NovaSeq platform. All reads produced by CUT&Tag were aligned to the mm10 mouse genome using Bowtie v1.1.1 with no more than two mismatches, and then only the uniquely mapped reads were used for peak calling analysis. MACS software was used for peak calling with default cutoffs (84). Peaks were assigned to the nearest genes using Homer. ChIPseeker was used to annotate the peaks. Total CTCF peaks and read density fold-change information based CUT-Tag-seq were provided in the Table. S4.

### Statistical analysis

Data were expressed as mean ± SEM (standard error of the mean). Unpaired Student’s *t* test was used for two-group comparisons. Comparison of means was performed using the independent-samples Student’s *t* test. In order to compare the differences of quantitative data between groups, normal distribution of data was verified and statistical analysis was carried out by analysis of variance (ANOVA). GraphPad Prism 7 software (GraphPad Software) was used for statistical analysis. A value *p* < 0.05 was used to determine significant difference. ∗ Indicates *p* < 0.05, ∗∗ indicates *p* < 0.01, ∗∗∗ indicates *p* < 0.001, and ∗∗∗∗ indicates *p* < 0.0001.

## Data availability

The RAN-seq, ATAC-seq, and CUT&Tag-seq data were deposited to the NCBI SRA database (SRP338348, SRP338500, and SRP338387). Other data were provided in the form of supplementary information.

## Supporting information

This article contains supporting information.

## Conflict of interest

The authors declare that they have no conflicts of interest with the contents of this article.
