# Supplementary material for: Transcriptional regulation of FACT involves Coordination of chromatin accessibility and CTCF binding
Source: J Biol Chem. 2023 Dec 10;300(1):105538. doi: 10.1016/j.jbc.2023.105538 (PMC10808957; doi:10.1016/j.jbc.2023.105538)
Supplement: Supporting Figures S1–S5 [file mmc5.docx]

**Transcriptional Regulation of FACT Involves Coordination of Chromatin Accessibility and CTCF Binding**

Peijun Wang^1,3,5#^, Na Fan^1,3#^, Wanting Yang^1^, Pengbo Cao^1^, Guojun Liu^5^, Qi Zhao^1, 2^, Pengfei Guo^1,2^, Xihe Li^1,4^, Xinhua Lin^2^, Ning Jiang^2*^& Buhe Nashun^1,3*^

^1^*Inner Mongolia Key Laboratory for Molecular Regulation of the Cell, Inner Mongolia University, Hohhot, 010070, China*

^2^*State Key Laboratory of Genetic Engineering, School of Life Sciences, Fudan University, Shanghai, 200438, China*

^3^*State Key Laboratory of Reproductive Regulation and Breeding of Grassland Livestock, School of Life Sciences, Inner Mongolia University, Hohhot, 010070, China*

*^4^Inner Mongolia Saikexing Institute of Breeding and Reproductive Biotechnology in Domestic Animals, Hohhot 011517, China*

*^5^School of Life Science and Technology, Inner Mongolia University of Science and Technology, Baotou 014010, China.*

*^#^ Contributed equally.*

** Correspondence:* [ningjiang@fudan.edu.cn](mailto:ningjiang@fudan.edu.cn) (N.J.)

** Correspondence:* [bnashun@imu.edu.cn](mailto:bnashun@imu.edu.cn) (B.N.)；*Tel:* +86 (0)471-4996885


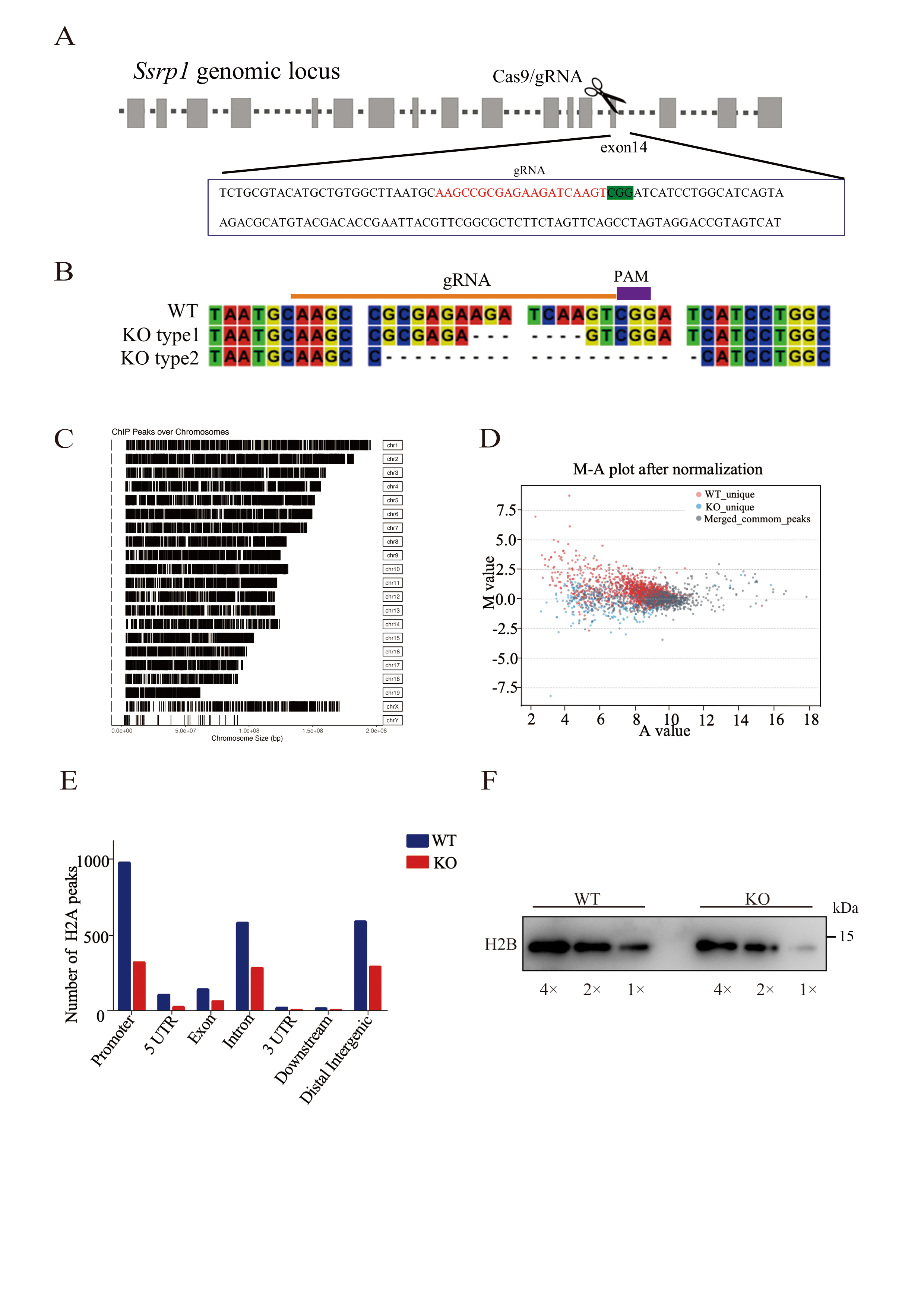


**Fig. S1 FACT deletion reduced chromatin content of histone H2A and H2B..**

(**A**)**:** Schematic illustration of the gRNA that targets exon 14 of the Ssrp1 gene. (**B**): Sanger sequencing of mutation sites in the two Ssrp1-KO cell clones. Dotted lines represent missing nucleotides, orange line indicates position of the gRNA, and purple box represents the PAM sequence. (**C**)**:** Chromosomal distribution of Ssrp1 CUT&Tag peaks in WT cells. (**D**)**:** MA plot showing histone H2A occupancy in WT and Ssrp1-KO cells. The x-axis represents averaged read density (log2 converted) of WT and Ssrp1-KO cells. The y-axis represents log2 fold change of normalized read densities between WT and Ssrp1-KO. (**E**)**:** Histogram shows genomic distribution of CUT&Tag peaks in the WT and Ssrp1-KO cells. Each bar represents the number of peaks in different genomic contexts. (**F**)**:** FACT deletion reduced histone H2B occupancy. Western blotting of acid extracted histone H2B at serial 1:2 dilutions starting from 5*10^4^ WT or Ssrp1-KO cells.


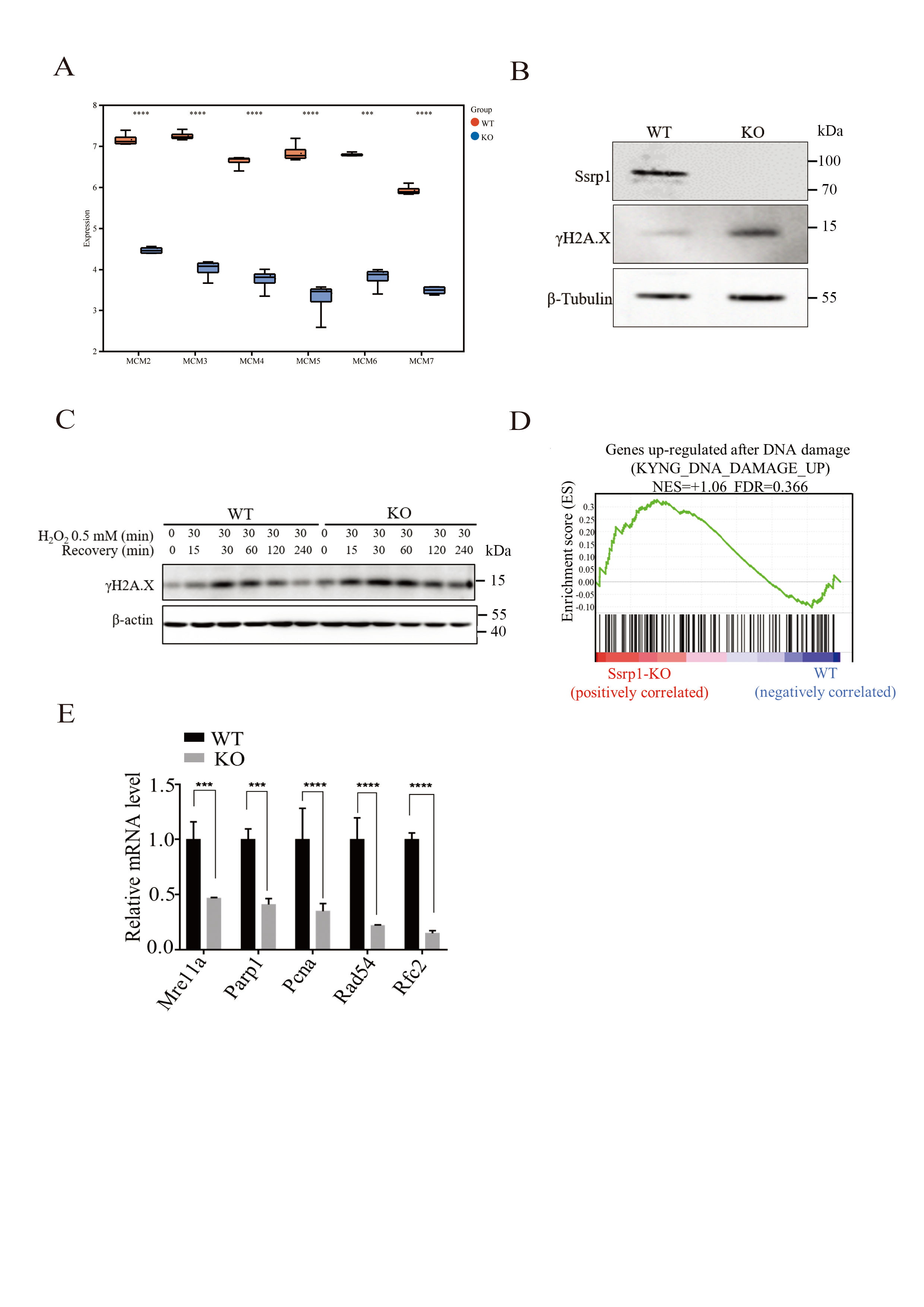


**Fig. S2 Loss of Ssrp1 impaired DNA replication and DNA damage repair.**

(**A**)**:** Boxplot showing expression of MCM2-7 (FPKM, obtained from RNA-seq) were significantly down-regulated in the Ssrp1-KO cells. (**B**)**:** Western blotting shows up-regulated γH2A.X levels in the Ssrp1-KO cells compared to the WT. (**C**)**:** Involvement of Ssrp1 in DNA repair process. WT and Ssrp1-KO cells were treated with 0.5 mM H_2_O_2_ in DMED+10% FBS for 30 min then allowed to recover in DMED+10% FBS for the indicated time periods prior to examination. γH2A.X was analyzed by western blotting and β-actin was used as internal control. (**D**)**:** Gene set enrichment analysis (GSEA) compares genes up-regulated after DNA damage (KYNG_DNA_DAMAGE_UP gene set) and the ranked list of genes expressed in the WT or Ssrp1-KO cells. NES, normalized enrichment score; FDR, false discovery rate. (**E**)**:** Ssrp1 deletion reduces the expression of DNA damage repair related genes. Expression of the indicated genes was analyzed by RT-qPCR and normalized against Gapdh. Data were obtained from three independent experiments and presented as mean ± SEM. Statistical analysis was carried out by the Student *t* test. ***P* < 0.01, ****P* < 0.001， or **** P < 0.0001.


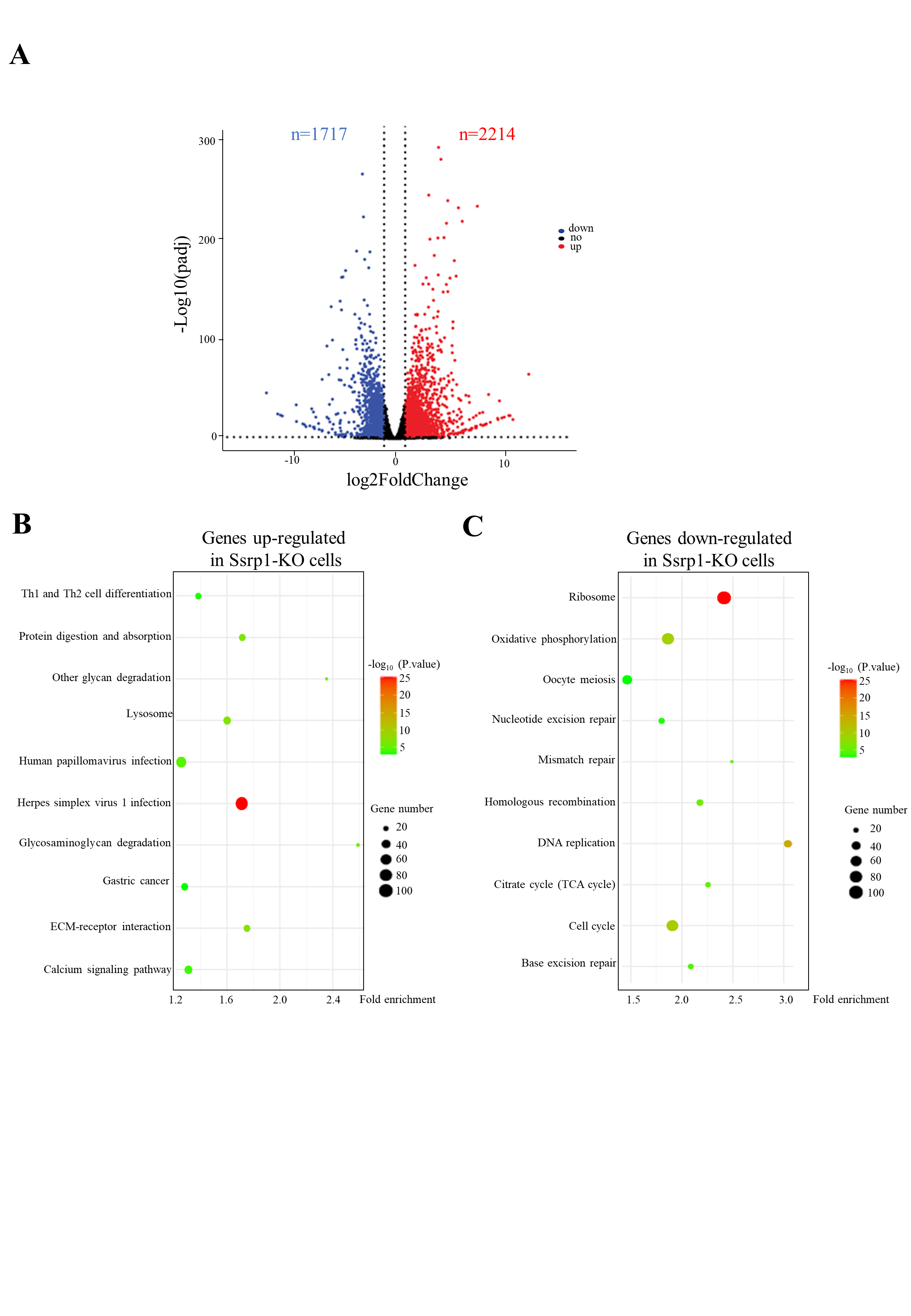


**Fig. S3 Analysis of the differentially expressed genes in WT and Ssrp1-KO cells.**

(**A**)**:** Volcano plot shows the differentially expressed genes identified in a pairwise comparison between WT and Ssrp1-KO cells. Up-regulated and down-regulated genes were colored in red and blue, respectively. (**B**): KEGG analysis of the up-regulated genes in Ssrp1-KO cells relative to WT cells**.** (**C**)**:** KEGG analysis of the down-regulated genes in Ssrp1-KO cells relative to WT cells.


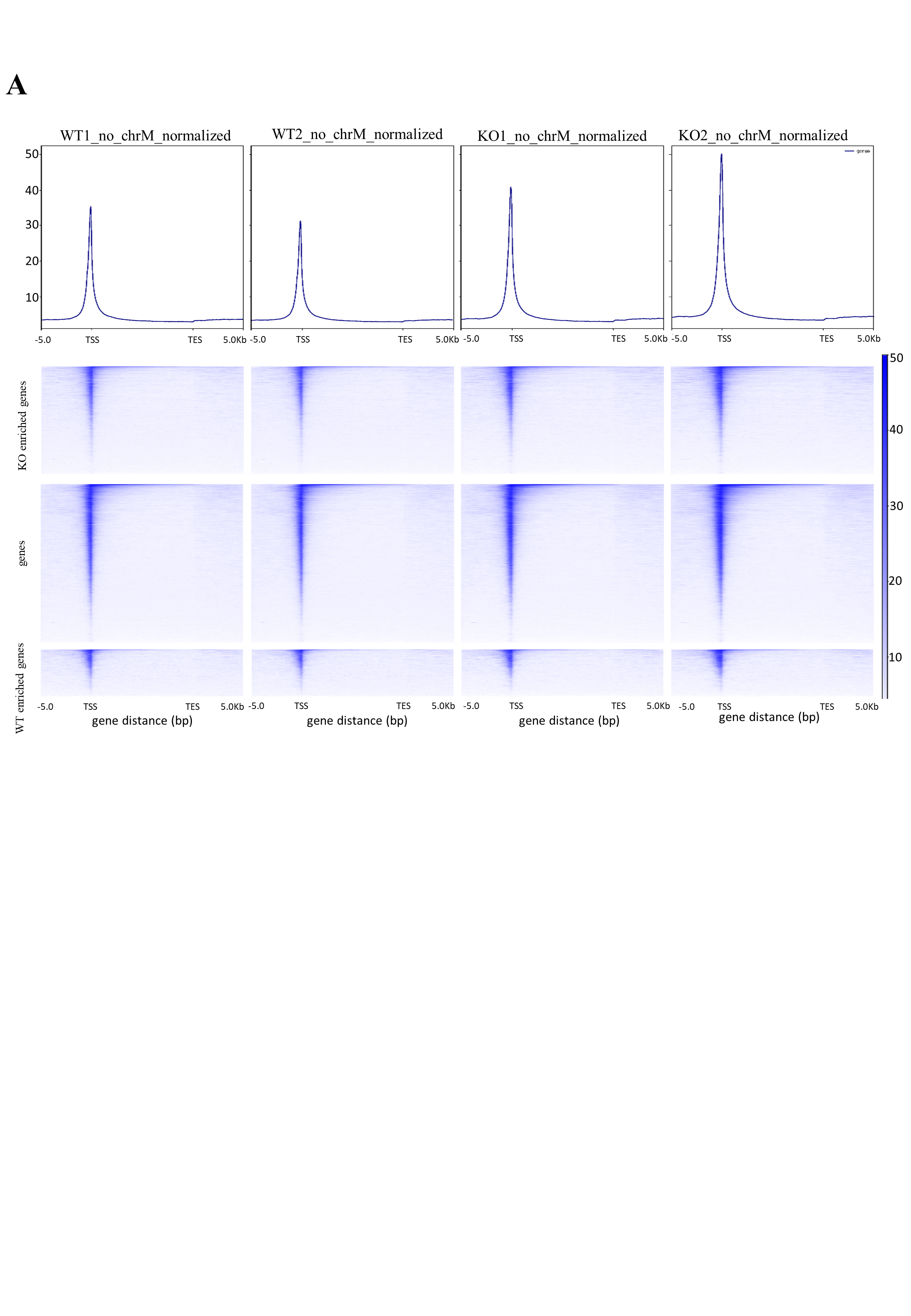


**Fig. S4 Disruption of Ssrp1 led to increased chromatin accessibility.**

(**A**)**:** Heatmap showing ATAC-seq peaks enriched around the TSS.


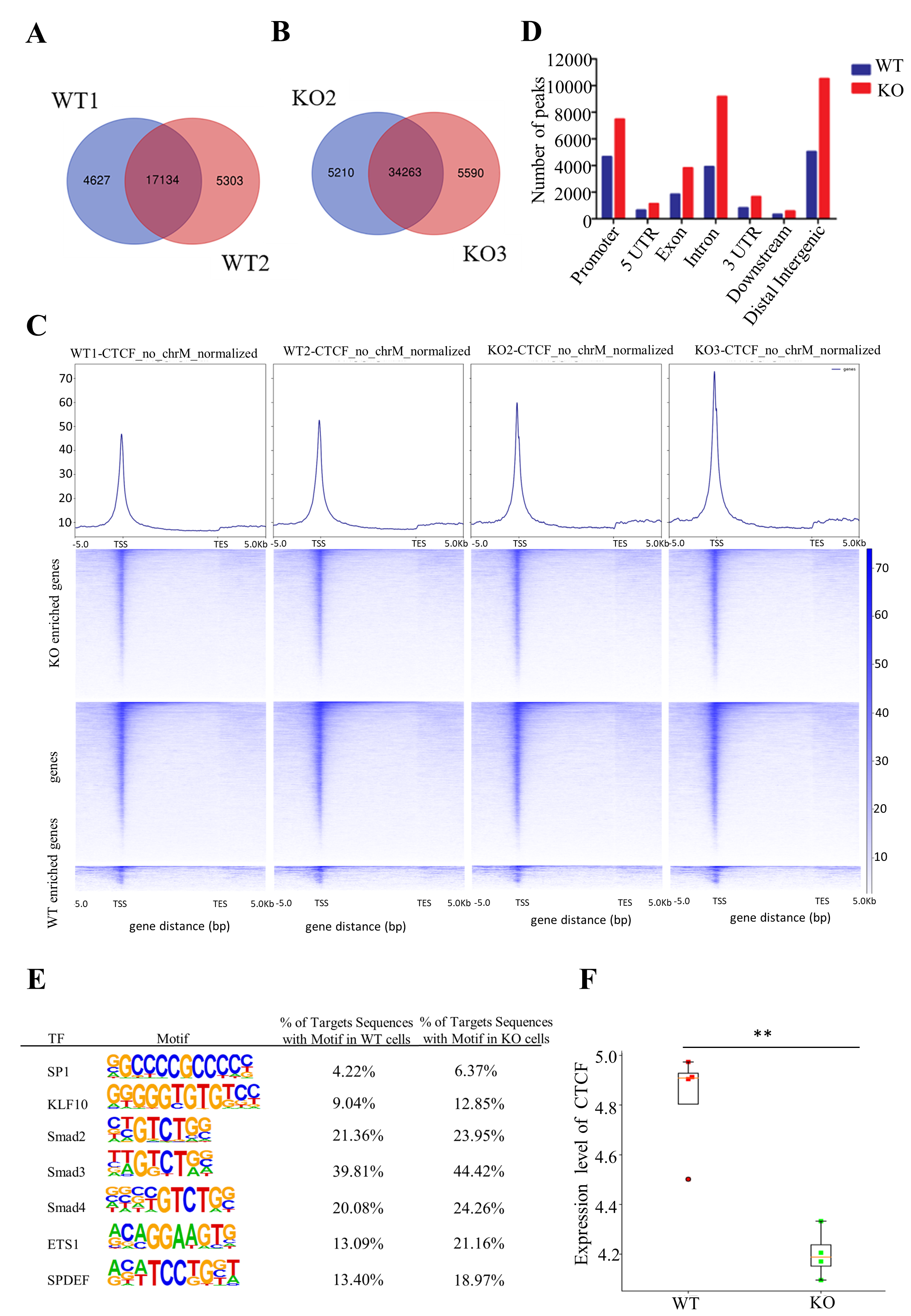


**Fig. S5 Chromatin binding of CTCF was significantly increased in the Ssrp1-KO cells.**

(**A-B**)**:** Venn diagram shows that CTCF CUT&Tag peaks in two biological replicates of WT (**A**) and Ssrp1-KO (**B**) cells have good consistency. (**C**)**:** Read density heatmap showing that CTCF CUT&Tag peaks are enriched around the TSS. (**D**)**:** Histogram shows genomic distribution of the CTCF CUT&Tag peaks in the WT and Ssrp1-KO cells. Each bar represents the number of peaks in different genomic contexts. (**E**)**:** Conserved motif analysis shows Ssrp1 deletion increases accessibility of GC-rich recognition motifs. GC-rich transcription factor recognition motifs and their proportions in the Ssrp1-KO and WT cells are shown. (**F**)**:** Boxplot representation of the RNA-seq data shows expression of CTCF was significantly down-regulated in the Ssrp1-KO cells.
